# Research on Decoupling Measurement Technology for 2-DOF Angular Signals Based on Spherical Capacitive Sensors

**DOI:** 10.3390/s26041215

**Published:** 2026-02-13

**Authors:** Shengqi Yang, Kezheng Chang, Zhipeng Zhang, Yaocheng Li, Yanfeng Liu, Zhong Li, Huiwen Wang

**Affiliations:** Northwest Institute of Mechanical & Electrical Engineering, Xianyang 712000, China

**Keywords:** spherical joint, 2-DOF angle measurement, capacitive sensor

## Abstract

As a core functional component of multi-degree-of-freedom precision motion mechanisms, spherical hinges are widely used in high-end equipment fields such as industrial robots, vehicle engineering, and intelligent manufacturing. Their dynamic performance directly determines the motion accuracy and the level of intelligent control of the equipment. The high-precision real-time measurement of two-degree-of-freedom (2-DOF) angles is a key prerequisite for achieving precise closed-loop control of spherical hinges. However, due to the strong coupling characteristics between the 2-DOF angle signals, it is difficult to directly and accurately measure the angular motion parameters of spherical hinges, which has become a core technical bottleneck restricting the improvement in their application efficiency. To address this challenge, this paper presents an improved study of the previously proposed spherical differential quadrature capacitance sensor for measuring the 2-DOF angle signals of spherical hinges. Firstly, the 2-DOF angle signal decoupling model is reconstructed and optimized. Secondly, a real-time decoupling circuit architecture for phase-shift detection with single-frequency signal excitation is innovatively proposed. This solution effectively addresses the incomplete decoupling of 2-DOF angle signals in previous studies, as well as the problems of considerable measurement noise, low resolution, and high calibration difficulty caused by random amplitude and phase errors in the excitation signals. Through the construction of an experimental platform for verification tests, the results show that the proposed scheme can significantly suppress the random errors caused by the parameter dispersion of the device, achieve an angle measurement resolution of 0.001°, and simultaneously considerably reduce the complexity of system calibration, laying a key technical foundation for the engineering application of spherical hinges in the fields of precision measurement and high-performance control.

## 1. Introduction

As key multi-degree-of-freedom motion elements, spherical hinges are of great engineering significance in high-end equipment applications. In industrial robotics, their multi-directional rotation characteristics enable high-precision tasks for robotic arms, such as precision assembly, welding, and material handling. In vehicle engineering, spherical hinges enhance the flexibility and dynamic stability of steering mechanisms by connecting transmission components, such as tie rods and suspension arms. In space exploration, spherical hinges enable multi-axis coordinated motion of robotic arms, enabling fine operations in extreme environments. With the increasing demand for flexibility and autonomy in intelligent equipment, spherical hinges are evolving toward precision mechatronic integrated systems, becoming core components in smart manufacturing. However, constrained by two-degree-of-freedom (2-DOF) coupling effects and nonlinear motion characteristics, traditional encoders and single-axis angle sensors struggle to achieve synchronous detection of the 2-DOF angle of the spherical joint. Therefore, developing real-time 2-DOF angle measurement technology based on novel sensing principles has become a key research direction to overcome the bottleneck in intelligent ball joint applications, with significant academic and engineering value.

In recent years, many scholars have conducted research on the 2-DOF angle measurement technologies for spherical hinges. Reference [1] proposed a 2-DOF angle measurement method based on the laser Doppler effect. This method uses a beam splitter to split the laser-generated horizontal beam into two beams: the reference beam I and the measurement beam II. By exploiting the Doppler effect that occurs when measurement beam II irradiates the spherical motor rotor, the frequency difference between the two beams is obtained. Analysis of this frequency difference enables the derivation of the 2-DOF angle of the spherical motor rotor.

References [2,3] introduced a 2-DOF angle measurement method based on a target tracking attitude estimation algorithm. This approach uses a high-speed camera to capture video of the spherical motor rotor’s 2-DOF angular motion. It applies an improved fast discriminant scale-space tracking (IFDSST) algorithm to process these sequences, thereby obtaining the 2-DOF angular position of the motor rotor.

These methods can be categorized as optical measurement techniques, which offer advantages such as high resolution and non-contact measurement. However, they suffer from limitations including complex optical paths, large system footprint, and poor environmental adaptability, making them unsuitable for the structural constraints of spherical hinges.

To address the shortcomings of optical methods, researchers have explored 2-DOF angle measurement technologies based on inertial devices. Reference [4] presented a 2-DOF angle measurement method using a micro-electro-mechanical system (MEMS) inertial sensor system. This method estimates the motion attitude and the 2-DOF angle of the spherical rotor using triaxial gyroscopes and accelerometers, while compensating for axial-angle errors to reduce inclination errors between the motor rotor and the MEMS sensor coordinate systems, thereby improving measurement accuracy. However, constrained by the inherent properties of inertial devices, the angle measurements exhibit significant time drift, failing to meet the operational duration requirements of spherical hinges.

To overcome this limitation, scholars have investigated 2-DOF angle measurement methods based on electromagnetic principles. Reference [5] proposed a three-degree-of-freedom (3-DOF) angle measurement method using a Hall sensor array comprising 64 sensors. This array detects magnetic field variations caused by the motor rotor’s rotation to determine the attitude of the rotor’s three rotational axes. Reference [6] introduced a method for measuring the 3-DOF angles of a permanent magnet spherical motor (PMSM) based on sensor data fusion. This approach simultaneously measures the magnetic flux density (MFD) field and back electromotive force (back EMF) using embedded sensors and estimates the 3-DOF angles of the PMSM through a Kalman filter (KF) sensor fusion algorithm. However, such methods face challenges, including susceptibility to small magnetic field fluctuations and interference that affect measurement accuracy, stringent requirements for sensor array positioning, and the introduction of Abbe errors from multiple sensor groups.

To address the limitations of electromagnetic measurement methods, researchers have developed 2-DOF angle measurement techniques based on eddy current sensors. Reference [7] proposed an attitude detection method based on stator coil mutual inductance, which employs artificial intelligence algorithms to analyze the relationship between stator/rotor magnetic pole misalignment angles and mutual inductance voltages to deduce the spherical rotor’s 3-DOF angular position. Reference [8] presented a method for measuring the 3-DOF angles of ball joints using eddy current sensors. This method generates two-dimensional absolute encoding using pseudo-random codes, maps it onto a spherical surface to create three-dimensional absolute encoding, and identifies the resulting encoding utilizing an array of eddy current sensors embedded in the ball socket. Reference [9] proposed a 2-DOF angle measurement method using eddy current sensors, where four sensors measure the ball head’s rotation angle and an extreme learning machine (ELM) artificial neural network algorithm calculates the 2-DOF angle signals.

Eddy current sensor-based measurement methods offer advantages such as strong anti-interference capability and compact size. However, they exhibit limitations, including complex angle feature recognition encoding with high manufacturing difficulty, significant temperature sensitivity leading to poor environmental adaptability, and software decoupling schemes based on AI algorithms that are susceptible to experimental factors, resulting in insufficient decoupling accuracy and robustness.

To address the shortcomings of eddy current sensors, researchers have proposed 2-DOF angle measurement methods based on capacitive sensors. Reference [10] arranged three spherical electrodes in the socket as driving electrodes and one on the ball head as the sensing electrode, with all four electrodes’ spherical centers coinciding at one point to form three variable-area capacitive sensors. Rotation of the spherical motion pair changes the electrode area, generating variable capacitance signals that enable the detection of 2-DOF angular motion. Reference [11] proposed a 3-DOF displacement measurement method based on a spherical capacitive grating sensor, which detects 3-DOF displacement signals from capacitance variations between grating electrodes during motion and employs AI algorithms to train on experimental data and inversely deduce the 3-DOF displacement. While this method effectively overcomes the temperature sensitivity issues of eddy current sensors, it still fundamentally suffers from Abbe errors introduced by multiple sensor electrodes, as well as the poor real-time performance and robustness of AI-based decoupling schemes. Therefore, it is necessary to further research the 2-DOF angle decoupling measurement scheme.

To address the aforementioned technical bottlenecks, an innovative scheme based on a spherical differential four-quadrant capacitive sensor is proposed [12,13], which has demonstrated the feasibility of this non-contact measurement method for 2-DOF angle detection. It has systematically quantified the nonlinear impact of installation errors on measurement accuracy x. However, proposed decoupling circuits exhibit random amplitude and phase errors, resulting in high system calibration complexity and difficulty meeting engineering application requirements.

In this study, after comprehensively considering the advantages and disadvantages of various 2-DOF angle signal decoupling measurement methods for spherical hinges, the spherical differential four-quadrant capacitive sensor was still selected as the core measurement device to research the 2-DOF angle signal measurement of spherical hinges. Building on this selection and in response to the inherent defects of the 2-DOF angle signal decoupling model identified in previous research, this paper focuses on optimizing it. It reconstructs a more accurate decoupling model and proposes a 2-DOF angle signal real-time decoupling circuit, which is suitable for the improved model. This improved scheme effectively addresses key problems identified in prior research, such as incomplete decoupling of 2-DOF angle signals and the introduction of random noise due to parameter discreteness across multiple devices. As a result, it significantly enhances the system’s measurement resolution while greatly reducing its calibration complexity.

Addressing the shortcomings in system accuracy caused by multi-device parameter dispersion and amplitude–phase errors in the four-channel excitation circuit, this study proposes an optimized decoupling scheme. By reconstructing the 2-DOF angular decoupling model, adjusting the excitation electrode polarity, and simplifying the signal acquisition and processing circuitry, the excitation signal is optimized from a four-channel, dual-frequency superimposed form to a single-channel sinusoidal excitation, while significantly reducing the number of analog components. Consequently, this approach fundamentally resolves problems encountered in previous research, such as difficulties in sensor initialization calibration and significant measurement noise, which stem from device parameter scattering and errors among the four-channel excitation signals. Experiments show that the improved scheme effectively suppresses random errors introduced by discrete device parameters, achieving a measurement resolution of 0.0017°. Additionally, temperature drift and nonlinear error are better than 0.00008°/°C and ±1.53%, respectively.

## 2. Materials and Methods

### 2.1. Mathematical Modeling of the 2-DOF Angle Measurement Method

**Figure 1:**
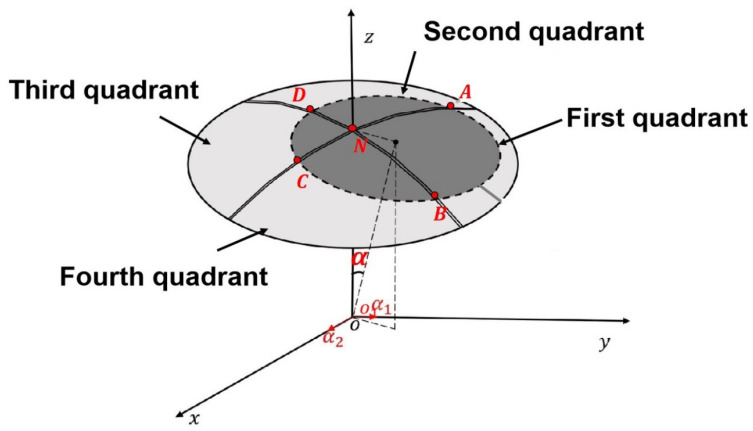
The schematic of the spherical capacitive sensor structure.

As shown in Figure 1, to detect the 2-DOF angle of the ball joint, a capacitive sensor based on a spherical differential four-quadrant electrode structure was designed, which consists of a spherical driving electrode A and a spherical sensing electrode B, with electrodes A and B concentrically arranged. The concave surface of electrode A is divided into four equal-area quadrant sections by insulating strips. Each part of electrode A forms a measurement capacitance with electrode B, denoted as $C_{1}$, $C_{2}$, $C_{3}$, and $C_{4}$, respectively. In the initial position, the areas between electrodes A and B are equal. Thus, the capacitances of $C_{1}$, $C_{2}$, $C_{3}$, and $C_{4}$ are balanced, and the differential capacitance signal output by the sensor is zero. When the ball joint rotates, it causes the areas between electrodes A and B to change, resulting in changes in the capacitance of the four measurement capacitors. At this time, the system outputs differential capacitance signals $\Delta C_{13}$ and $\Delta C_{24}$. By detecting the capacitance values of $\Delta C_{13}$ and $\Delta C_{24}$, the 2-DOF angle of electrode B around the origin *o* of the Cartesian coordinate system can be calculated. For the convenience of constructing subsequent angular measurement models and numerical analysis, the 2-DOF angle signals are decomposed into rotations around the *X*-axis and *Y*-axis, with the angle component around the *X*-axis denoted as $\alpha_{1}$ (Roll Angle) and the angle component around the *Y*-axis denoted as $\alpha_{2}$ (Pitch Angle).

Ignoring edge effects, parasitic capacitance, wiring resistance, and other factors, the derivation steps for the differential output capacitances $\Delta C_{13}$ and $\Delta C_{24}$ of the spherical capacitive sensor are as follows:

(1) $$\Delta C_{13}=\frac{\epsilon }{d_{0}}{\;\int \int \;}_{ANB}1d\sigma -\frac{\epsilon }{d_{0}}{\;\int \int \;}_{DNC}1d\sigma$$

(2) $$\Delta C_{24}=\frac{\epsilon }{d_{0}}{\;\int \int \;}_{\mathrm{DNA}}1\;d\sigma -\frac{\epsilon }{d_{0}}{\;\int \int \;}_{\mathrm{BNC}}1\;d\sigma$$

(3) $$\Delta C_{13}=\frac{\epsilon H}{d_{0}}\cdot \left[\arcsin\left(\frac{R}{r}\alpha_{1}\right)+\arcsin\left(\frac{R}{r}\alpha_{2}\right)\right]$$

(4) $$\Delta C_{24}=\frac{\epsilon H}{d_{0}}\cdot \left[\arcsin\left(\frac{R}{r}\alpha_{1}\right)-\arcsin\left(\frac{R}{r}\alpha_{2}\right)\right]$$

where

- *H* is $Rr\left(\pi -2\arccos\frac{r}{R}\right)$;
- ϵ is the permittivity, $\epsilon =\epsilon_{r}\epsilon_{0}$, where $\epsilon_{r}$ is the relative permittivity and $\epsilon_{0}$ is the vacuum permittivity;
- *d* is the zero-position gap between electrodes;
- $\alpha_{1}$ is the angular component of the gimbal rotation around the *X*-axis;
- $\alpha_{2}$ is the angular component of the gimbal rotation around the *Y*-axis;
- *R* is the radius of curvature of the convex spherical electrode;
- *r* is the projection radius of the convex spherical electrode.

According to practical applications, the measurement range of the capacitive sensor is within ±5°. Thus, the values of $\frac{R}{r}\alpha_{1}$ and $\frac{R}{r}\alpha_{2}$ are extremely small. Therefore, Equations (3) and (4) can be expanded using the Taylor series expansion as follows:

(5) $$\Delta C_{13}=\frac{\varepsilon H}{d_{0}}\left[\left(\frac{R}{r}\alpha_{1}\right)+\left(\frac{R}{r}\alpha_{2}\right)+\frac{1}{6}{\left(\frac{R}{r}\alpha_{1}\right)}^{3}+\frac{1}{6}{\left(\frac{R}{r}\alpha_{2}\right)}^{3}+\frac{3}{40}{\left(\frac{R}{r}\alpha_{1}\right)}^{5}+\frac{3}{40}{\left(\frac{R}{r}\alpha_{2}\right)}^{5}+\ldots \right]$$

(6) $$\Delta C_{24}=\frac{\varepsilon H}{d_{0}}\cdot \left[\left(\frac{R}{r}\alpha_{1}\right)-\left(\frac{R}{r}\alpha_{2}\right)+\frac{1}{6}{\left(\frac{R}{r}\alpha_{1}\right)}^{3}-\frac{1}{6}{\left(\frac{R}{r}\alpha_{2}\right)}^{3}+\frac{3}{40}{\left(\frac{R}{r}\alpha_{1}\right)}^{5}-\frac{3}{40}{\left(\frac{R}{r}\alpha_{2}\right)}^{5}+\ldots \right]$$

Thus, the differential output capacitances $\Delta C_{13}$ and $\Delta C_{24}$ of the capacitive sensor can be obtained when the sensing electrode rotates around the origin *O* into the first quadrant:

(7) $$\Delta C_{13}=\frac{\varepsilon HR}{rd}\cdot \left(\alpha_{1}+\alpha_{2}\right)$$

(8) $$\Delta C_{24}=-\frac{\varepsilon HR}{rd}\cdot \left(\alpha_{1}-\alpha_{2}\right)$$

By the same token, when the sensing electrode moves within the Cartesian coordinate system, the differential output capacitance of the capacitive sensor can be expressed by Equation (9):

(9) $$\Delta C_{4\times 4}=\frac{\varepsilon HR}{rd}\left[\begin{array}{cccc} \left(\alpha_{1}+\alpha_{2}\right) & \left(\alpha_{1}-\alpha_{2}\right) & -\left(\alpha_{1}+\alpha_{2}\right) & -\left(\alpha_{1}-\alpha_{2}\right)\\ \left(\alpha_{1}-\alpha_{2}\right) & \left(\alpha_{1}+\alpha_{2}\right) & -\left(\alpha_{1}-\alpha_{2}\right) & -\left(\alpha_{1}+\alpha_{2}\right)\\ -\left(\alpha_{1}+\alpha_{2}\right) & -\left(\alpha_{1}-\alpha_{2}\right) & \left(\alpha_{1}+\alpha_{2}\right) & \left(\alpha_{1}-\alpha_{2}\right)\\ -\left(\alpha_{1}-\alpha_{2}\right) & -\left(\alpha_{1}+\alpha_{2}\right) & \left(\alpha_{1}-\alpha_{2}\right) & \left(\alpha_{1}+\alpha_{2}\right) \end{array}\right]$$

$\Delta C_{4\times 4}\left[i,\;j\right]$ represents the sensor output capacitance value $\Delta C_{13}$ or $\Delta C_{24}$ when the gimbal moves into the *i*-th quadrant. For example, $\Delta C_{4\times 4}\left[1,\;1\right]$ represents the capacitance value of $\Delta C_{13}$ when the gimbal moves into the first quadrant, $\Delta C_{4\times 4}\left[1,\;2\right]$ represents the capacitance value of $\Delta C_{24}$ when the gimbal moves into the first quadrant, $\Delta C_{4\times 4}\left[2,\;1\right]$ represents the capacitance value of $\Delta C_{13}$ when the gimbal moves into the second quadrant, $\Delta C_{4\times 4}\left[2,\;2\right]$ represents the capacitance value of $\Delta C_{24}$ when the gimbal moves into the second quadrant, and so on.

From Equation (9), it can be seen that the 2-DOF angle signals $\left\{\alpha_{1},\alpha_{2}\right\}$ in the differential output capacitances $\Delta C_{13}$ and $\Delta C_{24}$ cannot be solved by individually detecting the capacitance values of $\Delta C_{13}$ or $\Delta C_{24}$.

The core objective of the decoupling model is to separate the coupled degrees, $\left\{\alpha_{1},\alpha_{2}\right\}$, in the differential capacitance signals, enabling independent output of the precise numerical values of each degree of freedom. Based on the analysis of the coupling characteristics mentioned above, this study designs a dedicated decoupling matrix $\delta^{\prime }$, which achieves the decoupling operation on the coupled signals by establishing a linear transformation relationship between $\Delta C_{13}$ and $\Delta C_{24}$, thereby separately calculating $\alpha_{1}$ and $\alpha_{2}$.

(10) $$\delta^{\prime }=\left[\begin{array}{cc} 1 & 1\\ 1 & -1\\ -1 & 1\\ -1 & -1 \end{array}\right]$$

The decoupling operation is shown in Equation (11):

(11) $$\frac{\varepsilon HR}{rd}\left[\begin{array}{cccc} \left(\alpha_{1}+\alpha_{2}\right) & \left(\alpha_{1}-\alpha_{2}\right) & -\left(\alpha_{1}+\alpha_{2}\right) & -\left(\alpha_{1}-\alpha_{2}\right)\\ \left(\alpha_{1}-\alpha_{2}\right) & \left(\alpha_{1}+\alpha_{2}\right) & -\left(\alpha_{1}-\alpha_{2}\right) & -\left(\alpha_{1}+\alpha_{2}\right)\\ -\left(\alpha_{1}+\alpha_{2}\right) & -\left(\alpha_{1}-\alpha_{2}\right) & \left(\alpha_{1}+\alpha_{2}\right) & \left(\alpha_{1}-\alpha_{2}\right)\\ -\left(\alpha_{1}-\alpha_{2}\right) & -\left(\alpha_{1}+\alpha_{2}\right) & \left(\alpha_{1}-\alpha_{2}\right) & \left(\alpha_{1}+\alpha_{2}\right) \end{array}\right]\cdot \left[\begin{array}{cc} 1 & 1\\ 1 & -1\\ -1 & 1\\ -1 & -1 \end{array}\right]$$

After the decoupling operation, the output of the system is given by Equation (12):

(12) $$\frac{\varepsilon HR}{rd}\left[\begin{array}{cc} 4\alpha_{1} & 4\alpha_{2}\\ 4\alpha_{1} & -4\alpha_{2}\\ -4\alpha_{1} & -4\alpha_{2}\\ -4\alpha_{1} & 4\alpha_{2} \end{array}\right]$$

As shown in Equation (12), the novel decoupling model proposed in this work successfully separates the originally coupled 2-DOF angle signals in the differential capacitances $\Delta C_{13}$ and $\Delta C_{24}$. This model effectively reduces mutual interference between signals, and its algorithmic structure is more amenable to hardware implementation. After processing by this model, the decoupling effect on the 2-DOF angle signals is more thorough, and no further calculation is required, thereby effectively overcoming the random errors introduced in existing research when calculating the 2-DOF angle signals.

### 2.2. Selection of Electrode Materials

The electrode materials are initially selected based on parameters such as the resistivity and temperature coefficient of the metallic materials. The relationship between resistivity and temperature coefficient can be expressed as follows:

(13) $$\rho \left(T\right)=\rho_{0}\left[1+\alpha (T-T_{0})\right]$$

where α is the temperature coefficient of resistivity, with units of 1/°C, and $\rho_{0}$ is the resistivity at temperature $T_{0}$.

Internationally, the conductivity of annealed copper (58MS/m) is often used as the standard conductivity (IACS, International Annealed Copper Standard) to calculate the relative conductivity of other materials.

From the parameters in Table 1, it can be seen that the relative electrical conductivities of gold, copper, and silver are 70.7% IACS, 103% IACS, and 109% IACS, respectively. All three are excellent conductors. Therefore, when these three materials are used to prepare electrodes, the electrodes can exhibit excellent electrical conductivity.

Further analysis of temperature stability shows that when the temperature varies by 100 °C, the resistance of the copper-based electrode only changes from 0.0006 Ω to 0.00083 Ω. This variation magnitude is much smaller than the inherent impedance of the sensor and will not exert an observable impact on its output characteristics.

Meanwhile, the temperature coefficients of resistivity of gold, silver, and copper show minor differences. Thus, when these three materials are adopted as electrode materials, the measurement errors induced by the temperature drift of electrode resistance can be neglected.

By comprehensively considering core indicators, including the electrical conductivity, ductility, corrosion resistance, and processability of electrode materials, gold is ultimately selected as the electrode material for the capacitive sensor in this study.

## 3. Design of Hardware Decoupling System

### 3.1. Hardware-Oriented Decoupling Method

**Figure 2:**
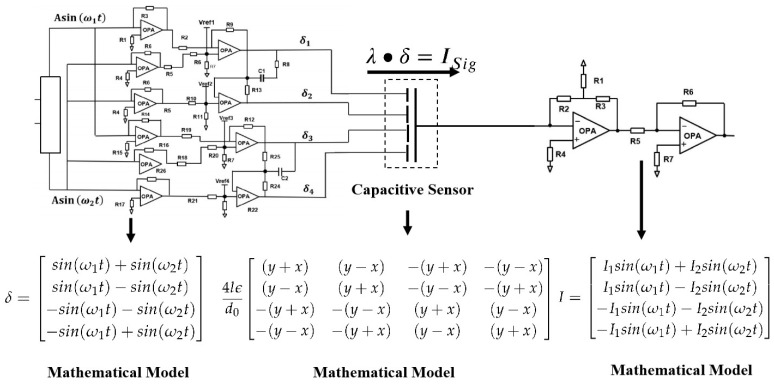
The hardware decoupling scheme with dual-frequency signal excitation.

A hardware decoupling scheme based on dual-frequency multiplexed excitation signals was proposed in previous research [12], with its decoupling principle illustrated in Figure 2.

Combined with previous research and Figure 2, discrete drift phenomena across multiple components in the driving circuit necessarily induce random amplitude and phase variations in the quad-channel dual-frequency excitation signals. Such imperfections substantially increase the complexity of subsequent system initialization and calibration procedures while concurrently degrading overall measurement precision.

**Figure 3:**
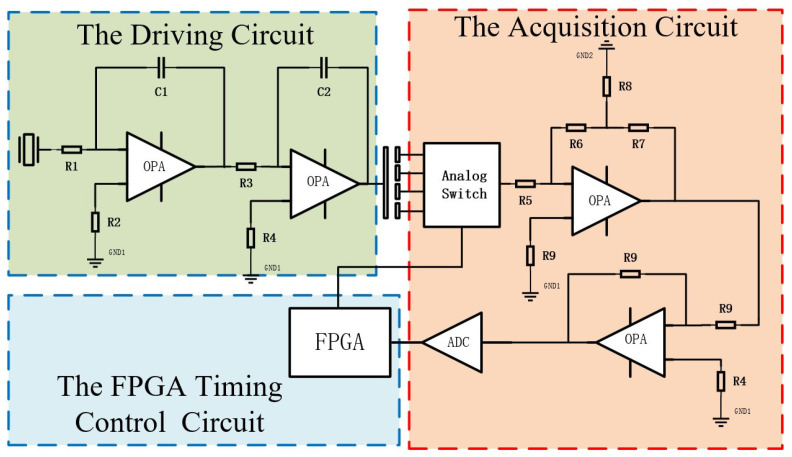
Block diagram of signal processing circuit scheme.

To mitigate the constraints imposed by multi-component parameter discreteness and inter-channel amplitude–phase errors in the four-channel excitation circuit on system precision, this study redesigned the signal acquisition and processing circuit based on a reconstructed decoupling model shown in Equation (10). The proposed design significantly reduces the number of analog components and simplifies the excitation scheme from a four-channel dual-frequency overlapping signal to a single-channel sinusoidal excitation signal. This fundamentally addresses the key technical challenges encountered in prior research. The circuit schematic is illustrated in Figure 3.

### 3.2. The Driving Circuit and Acquisition Circuit

The primary function of the driving circuit is to generate excitation signals for capacitive sensors, with its architecture based on an active crystal oscillator and a second-order integrating circuit configuration. The excitation sinusoidal signal is applied to the convex spherical electrode, facilitating capacitance-to-current (C-I) conversion for the capacitive sensor.

The primary function of the signal acquisition circuit is to capture weak current signals from sensors. This circuit consists of an analog switch, a T-type transimpedance amplifier, a second-stage amplification circuit, an analog-to-digital conversion circuit, and an FPGA-based timing control circuit. During operation, the analog switch, controlled by FPGA timing logic, sequentially routes the induced current signals from quadrant electrodes 1 through 4 to the T-type transimpedance amplifier for pre-amplification. Subsequently, the second-stage amplification circuit precisely amplifies the voltage signal from the transimpedance amplifier to match the ADC’s (16 bit, 4 MSPS) input range. Finally, the analog-to-digital conversion circuit converts the amplified signal to a digital format and transmits it to the FPGA for subsequent demodulation.

It should be noted that although coaxial cables can effectively shield the sensor system from electromagnetic interference, they will inevitably introduce parasitic capacitance. The driven cable technique can effectively eliminate the impact of the additional capacitance introduced by the coaxial shielded core wire on the sensor. However, this scheme imposes stringent requirements on the 1:1 amplifier, including phase shift, input capacitance (approaching 0), output current, and operating frequency band, which render its practical implementation rather challenging.

**Figure 4:**
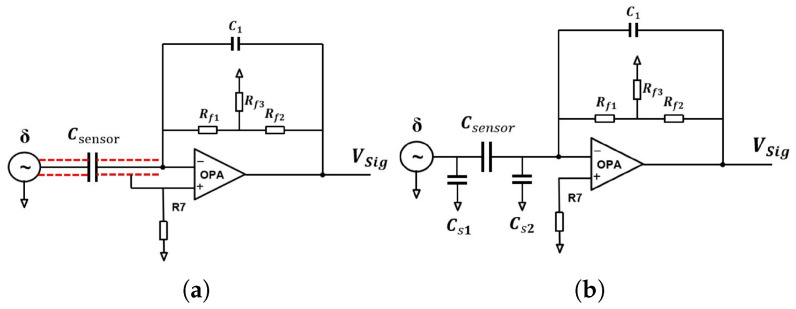
Design of T-type transconductance amplifier circuit: (**a**) schematic of equal cable driving technology and (**b**) equivalent diagram of cable stray capacitance.

To address this issue, as illustrated in Figure 4, the T-type transimpedance circuit proposed in this study leverages the operational amplifier’s “virtual short” and “virtual open” characteristics to maintain equipotentiality between the signal transmission path and the coaxial cable’s shielding layer. As a result, the equivalent capacitance of $C_{s2}$ is almost equal to 0 due to equal potentials at its two ends. Meanwhile, under AC drive conditions, the stray capacitance $C_{s1}$ is connected in parallel with the sensor capacitance $C_{\mathrm{sensor}}$ and thus has no impact on the detection accuracy. Ultimately, this achieves the goal of mitigating the influence of coaxial cable parasitic capacitance on the sensor.

### 3.3. FPGA Timing Control Circuit for Angle Signal Demodulation

**Figure 5:**
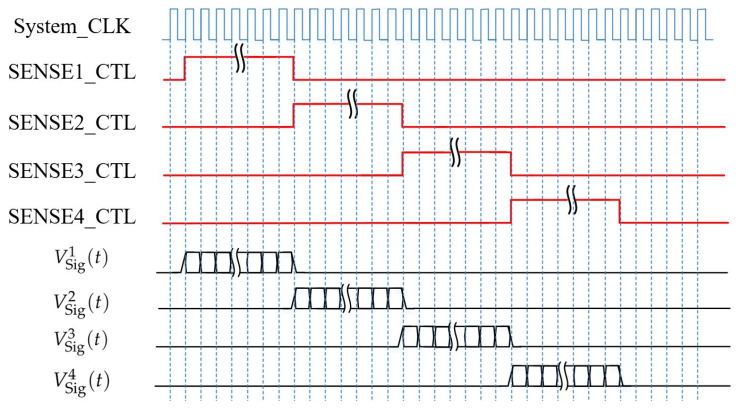
Timing control of the acquisition circuit.

**Figure 6:**
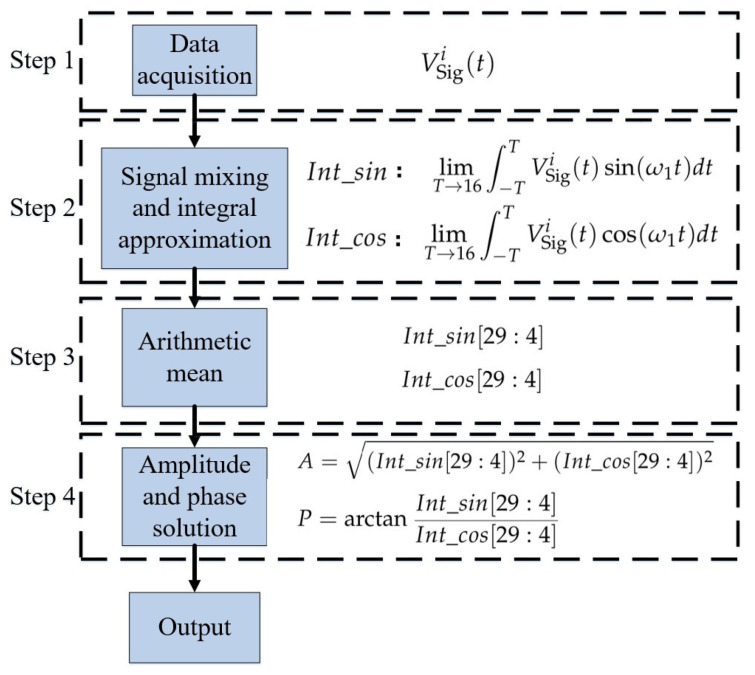
FPGA-based lock-in correlation demodulation process.

The core function of this module is timing control of analog switches in the acquisition circuit and demodulation processing of angle signals. The specific workflow is as follows: First, the FPGA accurately controls the on–off timing of high-speed analog switches to collect the induced output currents from the four-quadrant electrodes sequentially, and the corresponding timing logic is shown in Figure 5. Second, based on the lock-in cross-correlation algorithm, a cross-correlation operation is performed on the ADC sampling data, achieving the dual objectives of calculating the angle signal and significantly suppressing noise, as illustrated in Figure 6.

As is shown in Figure 5, $V_{\mathrm{Sig}}^{\left(i\right)}\left(t\right)$ is the output signal of the corresponding electrode and i∈{1,2,3,4} is the electrode index. For example, the output voltage signal from quadrant electrode 1 is $V_{\mathrm{Sig}}^{1}\left(t\right)$.

The FPGA cross-correlation calculation process is shown in Figure 6, with the specific design method described as follows:

- Reference signal generation: Two channels of digital reference signals are generated that are orthogonal and in phase with the signal to be calculated, with the same frequency and bit width.
- Signal mixing and integral approximation: The mixing operation is performed on the signal to be measured $V_{\mathrm{Sig}}^{i}\left(t\right)$ and on the two reference signal channels. Subsequently, the mixed digital signals are divided into groups—with the data of every 16 cycles (i.e., $2^{4}$ sampling points) as one group—and the grouped signals are accumulated to approximate the process of $R_{12}\left(\tau \right)=\lim_{T\to \infty }\int_{-T}^{T}S_{1}\left(t\right)\sin\left(\omega_{1}t\right)\;dt$ by using $\lim_{T\to 16}\int_{-T}^{T}V_{\mathrm{Sig}}^{i}\left(t\right)\sin\left(\omega_{1}t\right)\;dt$
- Arithmetic mean:Truncation processing is performed on Int_sin and Int_cos, and their arithmetic mean values, namely Int_sin[29:4] and Int_cos[29:4], can be obtained.
- Amplitude and phase solution: The quantitative solution of the amplitude and phase of $V_{\mathrm{Sig}}^{i}\left(t\right)$ is completed based on Euler’s formula.
  $$A=\sqrt{{\left(Int\_sin\left[29:4\right]\right)}^{2}+{\left(Int\_cos\left[29:4\right]\right)}^{2}}$$
  $$P=\arctan\frac{Int\_sin[29:4]}{Int\_cos[29:4]}$$

In this study, the cross-correlation calculation results are obtained by an approximation method. To this end, numerical analysis is used to compare the influence of approximation processes with different cycle numbers on the system’s calculation accuracy and data update rate, and the results are summarized in Table 2.

As shown in Table 2, using 16 data periods for the T→∞ process reconstruction achieves a good balance between accuracy loss and data update rate. This verifies the feasibility of this number of periods for the T→∞ process simulation.

## 4. Results

### 4.1. Design of the Experimental Setup

**Figure 7:**
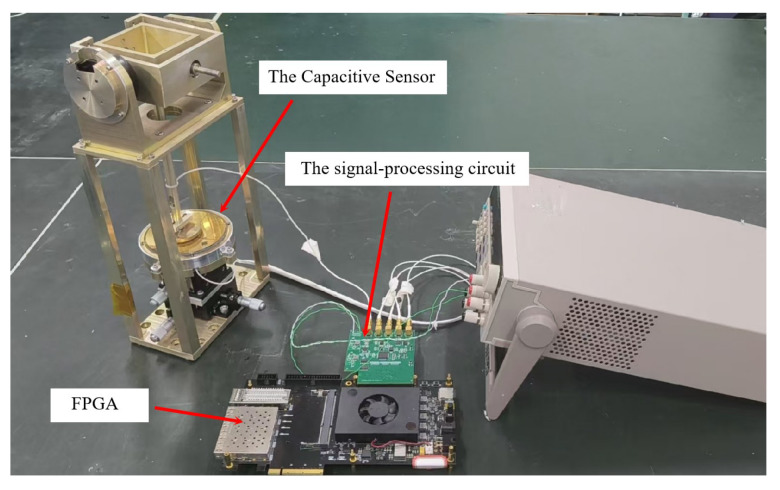
The spherical differential four-quadrant capacitive sensor experiment.

A prototype was constructed based on the scheme illustrated in Figure 7 for validation testing. The experimental setup comprises an electrode set, a 3-DOF displacement stage, drive motors, encoders, and hardware, among other components. It allows for validating single-angle measurement by locking a single axis or for validating 2-DOF angle measurement by simultaneously driving both axes, thereby enabling a comprehensive evaluation of the sensor’s precision measurement capabilities.

The details regarding electrode material selection, machining processes, electrical treatment procedures, assembly conditions, and assembly sequences have been elaborated in previous studies and will not be repeated herein [13].

### 4.2. Experiment on Two-Degree-of-Freedom Angle Measurement

This subsection aims to test the 2-DOF angle measurement performance of the designed prototype for spherical hinges. Three measurement schemes are specifically designed: the single-axis oscillation measurement experiment, the biaxial oscillation measurement experiment around the y=−x axis, and the biaxial oscillation measurement experiment around the y=2x axis. Among these schemes, the single-axis oscillation measurement experiment corresponds to a special motion attitude of the spherical hinge. In this state, the angle signal from one axis of the sensor is zero, and the core purpose of this experiment is to verify the sensor’s ability to independently measure the rotation angle of one axis when the other axis is fixed. The measurement experiment around the line y=−x also corresponds to a special motion attitude of the spherical hinge; in this state, the angle signals output by the two axes of the sensor are equal in value, and the experiment can fully characterize the sensor output characteristics when the driving electrode moves within the first to third quadrants. The measurement experiment around the line y=2x is designed to characterize the regular motion attitude of the spherical hinge. This motion mode not only facilitates position calibration of the pitch and roll motors but also effectively represents the sensor output when the driving electrode moves within the second to fourth quadrants. Based on the above characteristics, the latter two experiments are mainly used to jointly verify the sensor’s comprehensive measurement performance for the 2-DOF angle signals of the spherical hinge. In addition, the compensation and correction model for installation errors has been established in previous research [13]. Therefore, all sensor output data in this subsection have been compensated and corrected using that model.

#### 4.2.1. Measurement Experiment of Single-Axis Oscillation of Spherical Electrodes

In this experiment, only the pitch axis motor was driven to make the driving electrode oscillate reciprocally within the roll range of ±5° at a speed of 8°/s starting from 0°, while the roll axis remained stationary.

**Figure 8:**
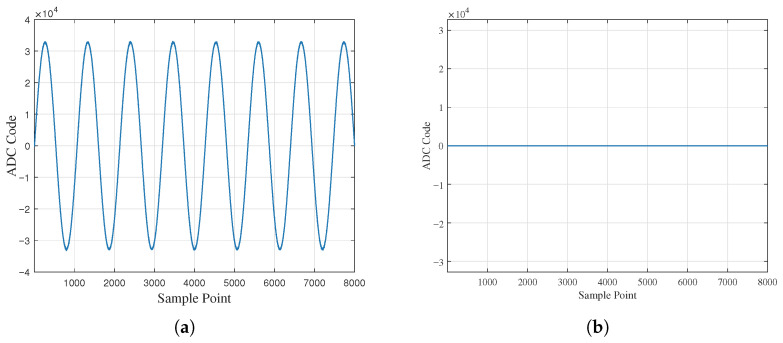
System output signals for single-DOF oscillation of the driving electrode: (**a**) pitch axis angle signal value and (**b**) roll axis angle signal value.

Based on the analysis of Figure 8, it can be observed that the angular motion output signal of the roll axis is zero at the initial position. The output of the pitch axis varies in a “sinusoidal” manner following the driving electrode, which is consistent with the motion trend of the driving electrode under these experimental conditions. Although the output curves of the pitch axis and roll axis in Figure 8 exhibit slight “glitch”-like fluctuations, such fluctuations fall within the scope of normal peak-to-peak ($V_{pp}$) noise. The relevant noise characteristics will be further analyzed in the subsequent section on resolution estimation.

#### 4.2.2. Measurement Experiment of Biaxial of Spherical Electrodes Around the Y=−X and Y=2x Axes

**Figure 9:**
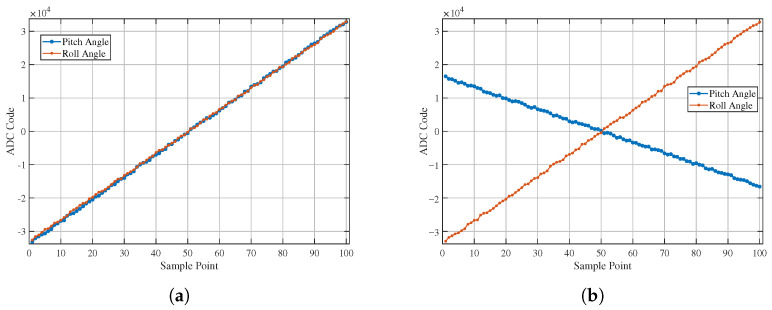
Measurement experiment of sensor 2-DOF measurement: (**a**) rotation experiment of drive electrode around line y = −x and (**b**) rotation experiment of drive electrode around line y = 2x.

The pitch and roll motors are synchronously driven to move the driving electrode to rotate around the line y=−x from the position (−5°, −5°) in the third quadrant to the position (5°, 5°) with a motion step of 0.1°; the output curves of the spherical capacitive sensor are shown in Figure 9a. The pitch and roll motors are driven synchronously to move the driving electrode to rotate around the line y=2x, with the motion step of 0.1°, from the position (−5°, 2.5°) in the second quadrant to the position (5°, −2.5°) in the fourth quadrant; the output curves of the spherical capacitive sensor are shown in Figure 9b.

The following can be seen from Figure 9:

- The variation trend of the 2-DOF angle signals output by the sensor is entirely consistent with the preset spatial motion trajectory of the driving electrode, which indicates that the designed spherical capacitive sensor can accurately detect the angular motion signals of the sensing electrode when it moves within the first to third quadrants and the second to fourth quadrants.
- There exists an obvious nonlinear error between the output data and the indicated values of the encoder, accompanied by “glitch” noise. Numerical analysis results show that the causes of the aforementioned errors and noise mainly include three aspects: first, the compensation and correction for electrode installation errors are insufficient, and the residual errors still affect the measured data; second, the FPGA-based digital phase-locked correlation algorithm experiences precision loss and the ADC sampling precision is limited; third, the poor control precision of the motor drive leads to the failure of the motion step size to meet the precise control requirement of 0.1°.

### 4.3. Estimation of Performance Parameters of the Experimental Prototype

#### 4.3.1. Nonlinear Error Estimation

There exists a significant nonlinear error between the output data and the theoretical values. Herein, the nonlinear error is used to characterize the degree of agreement between the sensor’s input–output characteristic curve and the ideal fitting straight line, and its calculation formula is given by

(14) $$e_{f}=\pm \frac{\Delta U_{max}}{U_{Fs}}\times 100\%$$

Here, $\Delta U_{\max}$ is the maximum deviation between the measured curve and the fitted straight line and $U_{Fs}$ is the full-scale output value of the sensor. It can be seen from Figure 9 that $\Delta U_{\max}$ is 537(ADC Code). Therefore, the nonlinear error is estimated as $e_{f}=\pm 1.53\%$.

#### 4.3.2. Resolution Estimation

The resolution of the capacitive sensor can be estimated based on the $V_{\mathrm{pp}}$ noise and sensitivity of the output data, and its calculation formula is given by

(15) $$R_{es}=\frac{\sigma }{S}$$

Herein, σ corresponds to the $V_{\mathrm{pp}}$ error in the data calculation process and *S* represents the sensitivity of data variation.

To accurately estimate the system resolution, this subsection selects the working condition in which the driving electrode rotates at a specified angle around the y=−x axis for analysis. In this condition, the variation trends of $\alpha_{1}$ and $\alpha_{2}$ are consistent, and the measurement resolution estimated using the rotation data under this condition is more representative. Meanwhile, to verify the stability of the system output data, the test duration is set to 1 h under these working conditions.

**Figure 10:**
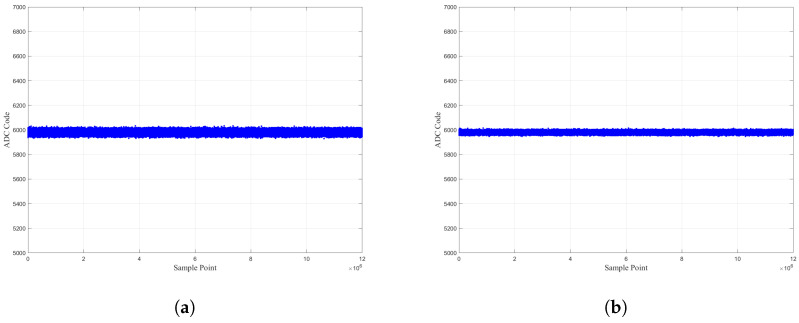
System resolution estimation: (**a**) pitch axis angle signal value and (**b**) roll axis angle signal value.

As shown in Figure 10, the $V_{\mathrm{pp}}$ noise of the angular motion signal $\alpha_{1}$ is 11×0.12=1.32 mV, and that of $\alpha_{2}$ is 9×0.12=1.08 mV. According to Equation (15), the measurement resolutions of $\alpha_{1}$ and $\alpha_{2}$ are, respectively,

(16) $$R_{es1}=\frac{1.32\;\mathrm{mV}}{0.8\;V/{}^{\circ}}=0.00165{}^{\circ}$$

(17) $$R_{es2}=\frac{1.08\;\mathrm{mV}}{0.8\;V/{}^{\circ}}=0.00135{}^{\circ}$$

#### 4.3.3. Environmental Adaptability Experiment

This subsection focuses only on the high- and low-temperature test items within the environmental tests. In the temperature characteristic test experiment, the specific operation procedure is as follows: first, rotate the induction electrode by 0.1° along the roll direction and keep it stationary; second, set the parameters of the high–low-temperature test chamber to increase the ambient temperature of the capacitive sensor from −30 °C to 50 °C at a gradient of 10 °C/step, with a holding time of 60 min at each temperature node; and finally, carry out three groups of parallel experiments repeatedly and record the output data of each experimental group at different temperature nodes.

**Figure 11:**
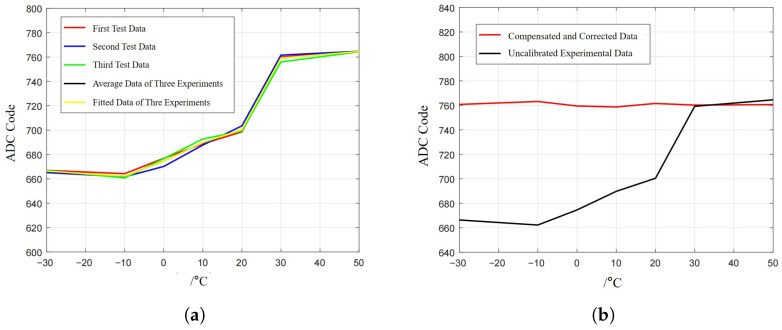
High–low-temperature operating performance test: (**a**) temperature drift curve of capacitive sensor and (**b**) temperature drift compensation and correction.

It can be seen from Figure 11a that the output temperature drift of the sensor is relatively large. However, according to the correlation characteristics between conductivity and temperature, when the temperature changes by 100 °C, the lead resistance $R_{\mathrm{wire}}$ of the electrode and coaxial cable changes from 0.12 Ω to 0.16 Ω, with a minimal variation magnitude; moreover, the resistance value of 0.16 Ω is far lower than the impedance of the capacitive sensor itself. Therefore, the temperature drift of the lead resistance of the coaxial cable and capacitive electrode has a negligible impact on the measurement results. In addition, the thermal expansion coefficient of the electrode material is approximately $14.2\times 10^{-6}/{}^{\circ}C$, and that of the substrate material is about $0.5\times 10^{-6}/{}^{\circ}C$. The resulting capacitance variation also exerts a negligible influence on the measurement results.

The temperature drift characteristics of various electronic devices in the signal processing circuit are relatively complex. The temperature drift coefficients of components such as resistors, capacitors, operational amplifiers, and crystal oscillators vary, making it difficult to achieve accurate theoretical calculations using specific mathematical methods.

Therefore, to reduce the influence of temperature variation on the output characteristics of the sensor, the least-squares method is first adopted to fit the average value of the three groups of experimental data in Figure 11a; second, an inverse function with respect to the parameter *T* (/°C) is constructed based on the fitting results, and this inverse function is used to perform reverse nonlinear correction on the sensor output to achieve the purpose of reducing the interference of temperature variation on the measurement data; and finally, the coefficients of the constructed inverse function are stored in the FPGA ROM to realize real-time correction of the sensor output results. As shown in Figure 11b, after correction, the temperature drift of the sensor is reduced from 0.001°/°C to 0.00008°/°C.

## 5. Discussion

Aiming to address the drawbacks of the previous spherical differential four-quadrant capacitive sensor, namely, incomplete decoupling of the decoupling model and random amplitude–phase errors in the signal processing circuit, this study proposes a novel 2-DOF angular reconstruction decoupling scheme. The decoupling model and signal processing circuit have been redesigned and optimized, and the effectiveness of the proposed scheme has been verified by experiments based on a test prototype. The experimental results indicate that installation errors remain in the system measurement data; meanwhile, the measurement resolution of the sensor is a theoretically estimated value, and a comprehensive evaluation of the system’s actual resolution and overall measurement accuracy has not been achieved. The reason lies in the fact that the simultaneous testing and calibration scheme for the resolution and accuracy of the 2-DOF angle of the spherical hinge is rather complex, and the relevant technical approaches are still under exploration by the research group. Therefore, future research will focus on developing high-precision calibration methods, resolution verification technologies, and installation error correction technologies for the 2-DOF angular signals of spherical hinges.

## 6. Conclusions

This study first summarizes the advantages and disadvantages of existing decoupling measurement methods for the 2-DOF angular motion of ball joints, as is shown in Table 3. It clarifies the distinct technical advantages of the spherical capacitive sensor scheme over other alternative technologies. Second, it optimizes the design of the 2-DOF angular decoupling scheme and the matching signal processing circuit for the spherical differential four-quadrant capacitive sensor. Subsequently, a test prototype is built to conduct experimental verification on the reconstructed decoupling model and signal processing circuit. The results demonstrate that the proposed scheme achieves complete decoupling of the 2-DOF angular signals of ball joints and effectively suppresses the random amplitude–phase noise in the signal processing circuit. Experimental results demonstrate that the angular resolution of the system is better than 0.0017° (improved by 15%). In contrast, the newly added test indicators, namely temperature drift and nonlinear error, are better than 0.00008°/°C and ±1.53%, respectively.

## Figures and Tables

**Table 1:** The resistivity of major metals and alloys at 20 °C.

Material	ρ ($10^{-8}\Omega \cdot m$)	σ (Ms/m)	IACS (%)	α (ppm/°C)
Silver	1.59	63	109	3800
Copper	1.68	59.6	103	3862
Gold	2.44	41	70.7	3840
Manganin	48.2	2.07	3.57	2
Constantan	49	2.04	3.52	8

**Table 2:** Impact of period number on precision loss and update.

Period (T)	Precision Loss (/°)	Update Rate (/KHz)
16	0.00054	3.125
128	0.00041	0.39
1024	0.00027	0.05

**Table 3:** Comparative analysis of performance metrics for typical decoupling measurement methods.

Decoupling Methods	Performance Advantages	Limitations
Optical method	High accuracy High resolution	Complex system structure High space occupancy Poor environmental adaptability
**Decoupling method** **based on AI algorithms**	**Simple architecture**	**Low decoupling accuracy** **Insufficient system robustness**
Multi-source measurement method	Direct measurement	Multi-source error accumulation
**Hardware decoupling method** **based on four-channel** **excitation signals** **(previous research)**	**Real-time decoupling**	**Introduce random** **amplitude–phase errors** **Great difficulty in initial calibration**
Hardware decoupling method based on single-channel excitation signal (this research)	Real-time decoupling Complete decoupling	Small angle measurement range (only ±5°)

## Data Availability

The data of 2-DOF angular signal measurements, sensor calibration records supporting the findings of this study are openly available in in Zenodo at https://doi.org/10.5281/zenodo.18344264.
